# Supplementary material for: Comparison of Change of Direction Speed Performance and Asymmetries between Team-Sport Athletes: Application of Change of Direction Deficit
Source: Sports (Basel). 2018 Dec 12;6(4):174. doi: 10.3390/sports6040174 (PMC6315619; doi:10.3390/sports6040174)
Supplement: Supplementary file 1 [file sports-06-00174-s001.zip › supplementary/Figure S1 - Scatter plots-v2.pdf]

<u>Figure S1. Spearman's correlations between 505 time, COD deficit, and 10-m sprint time for D and ND directions (pooled data, n =115)</u>

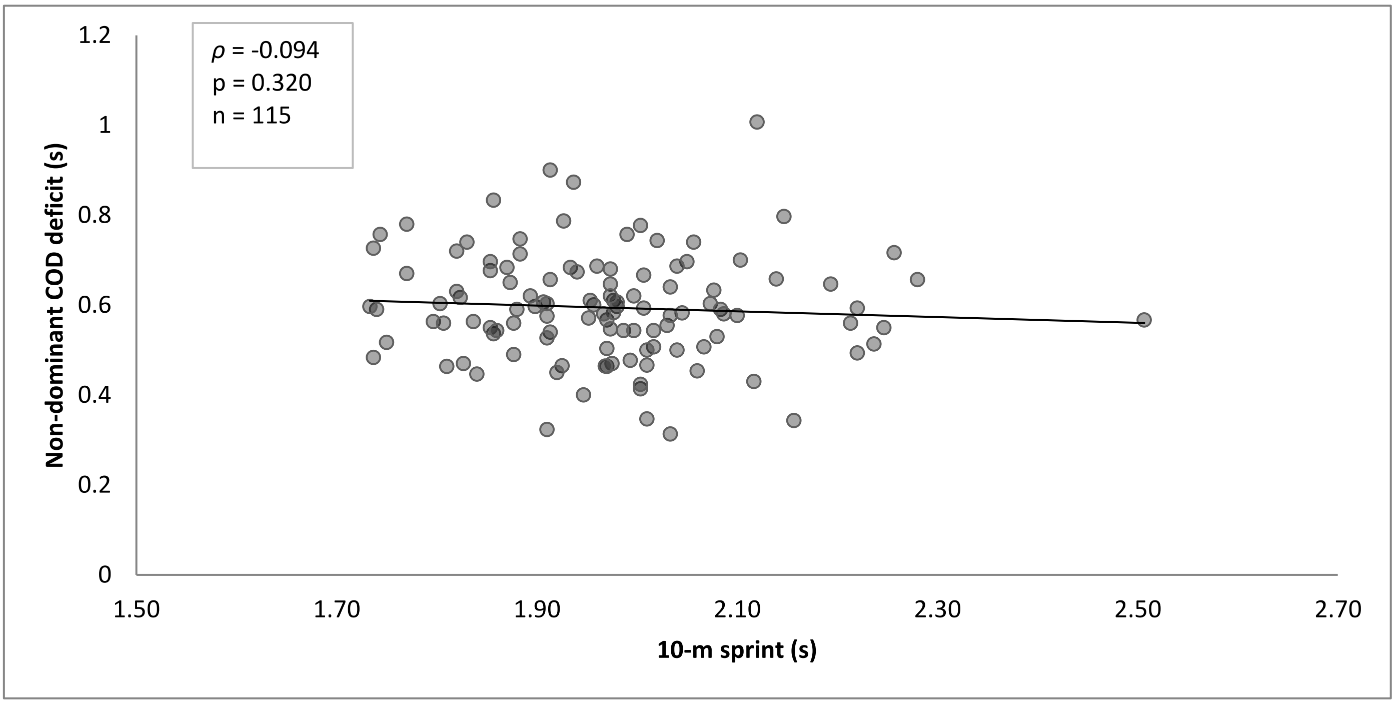

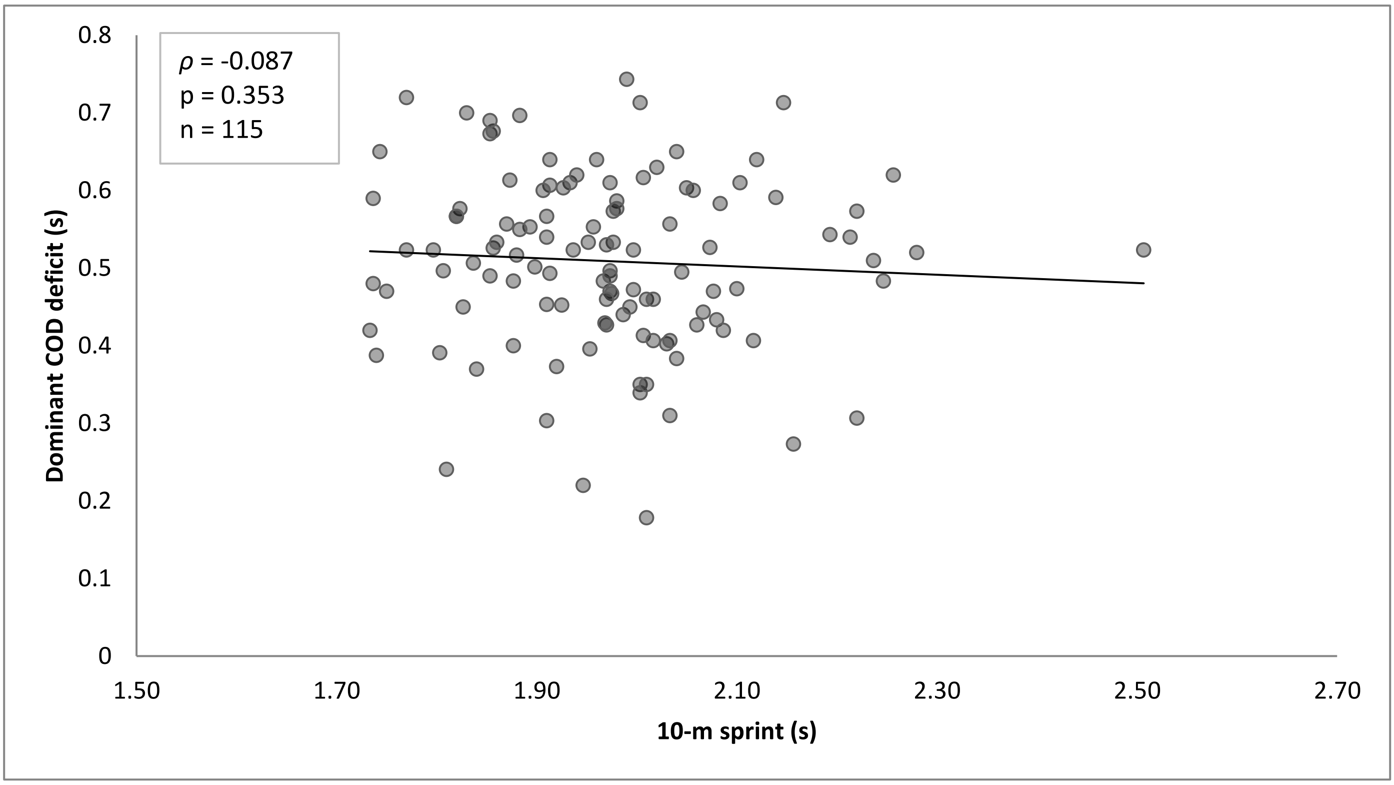

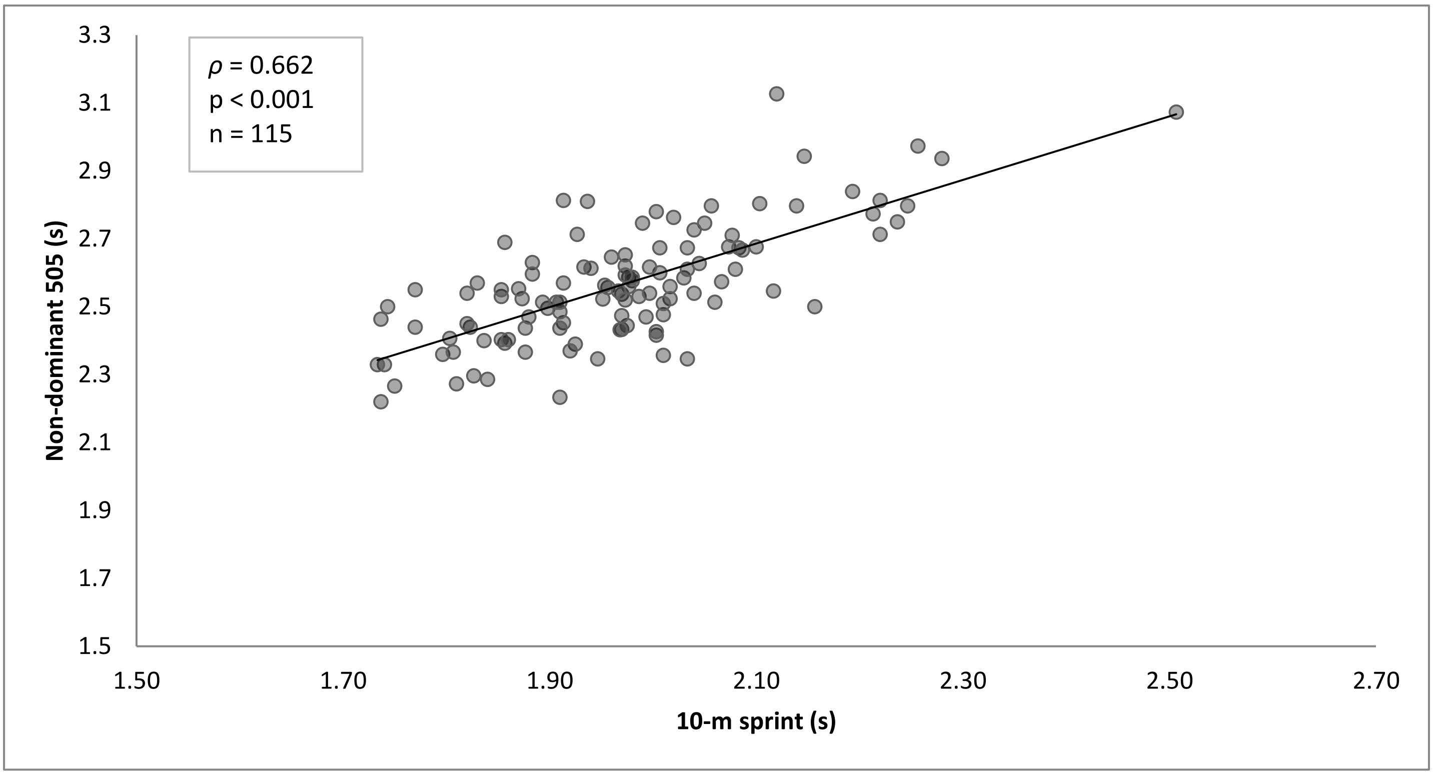
ρ = 0.662
p < 0.001
n = 115
Non-dominant 505 (s)
10-m sprint (s)
3.3
3.1
2.9
2.7
2.5
2.3
2.1
1.9
1.7
1.5
1.50
1.70
1.90
2.10
2.30
2.50
2.70

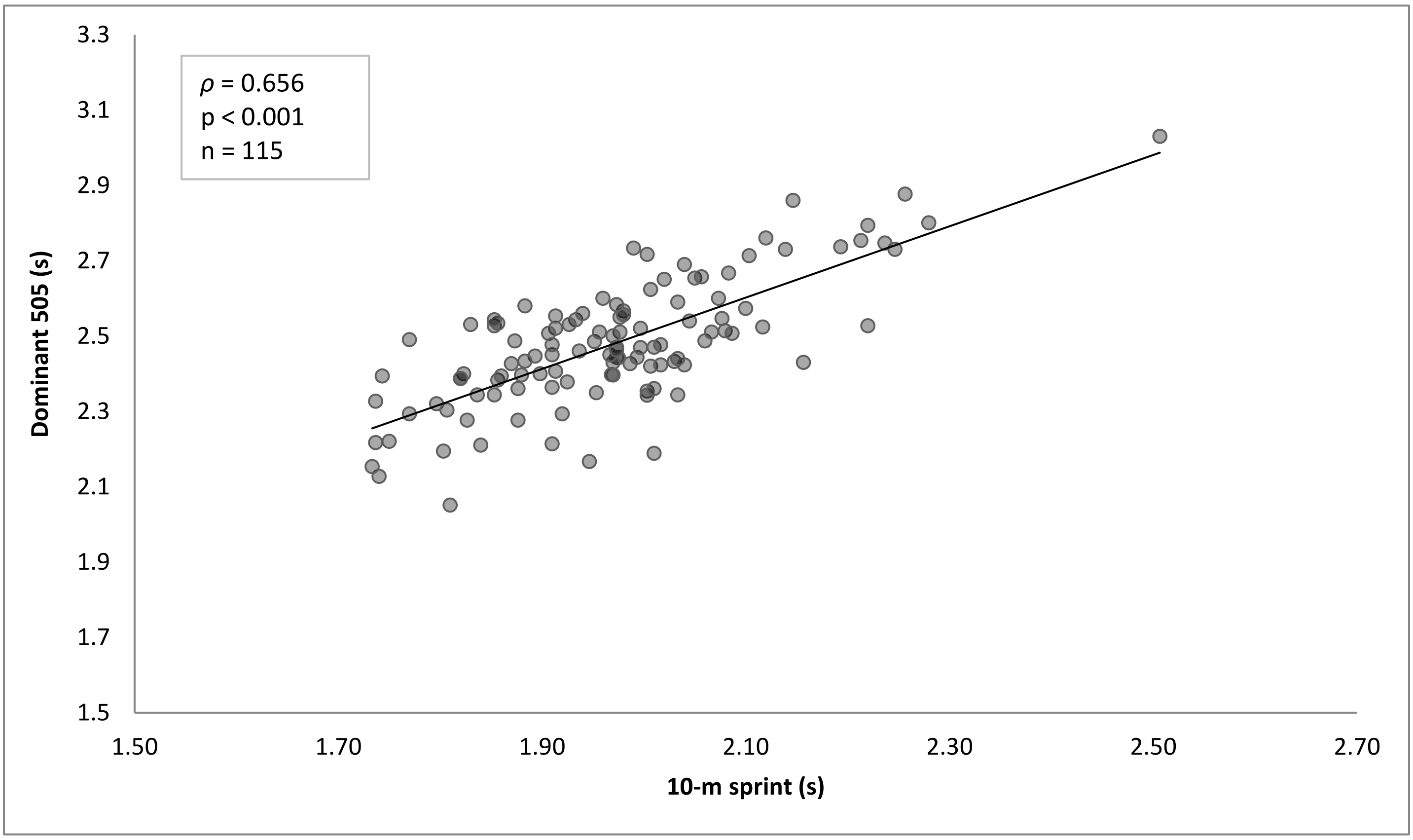
ρ = 0.656
p < 0.001
n = 115
Dominant 505 (s)
10-m sprint (s)
3.3
3.1
2.9
2.7
2.5
2.3
2.1
1.9
1.7
1.5
1.50
1.70
1.90
2.10
2.30
2.50
2.70

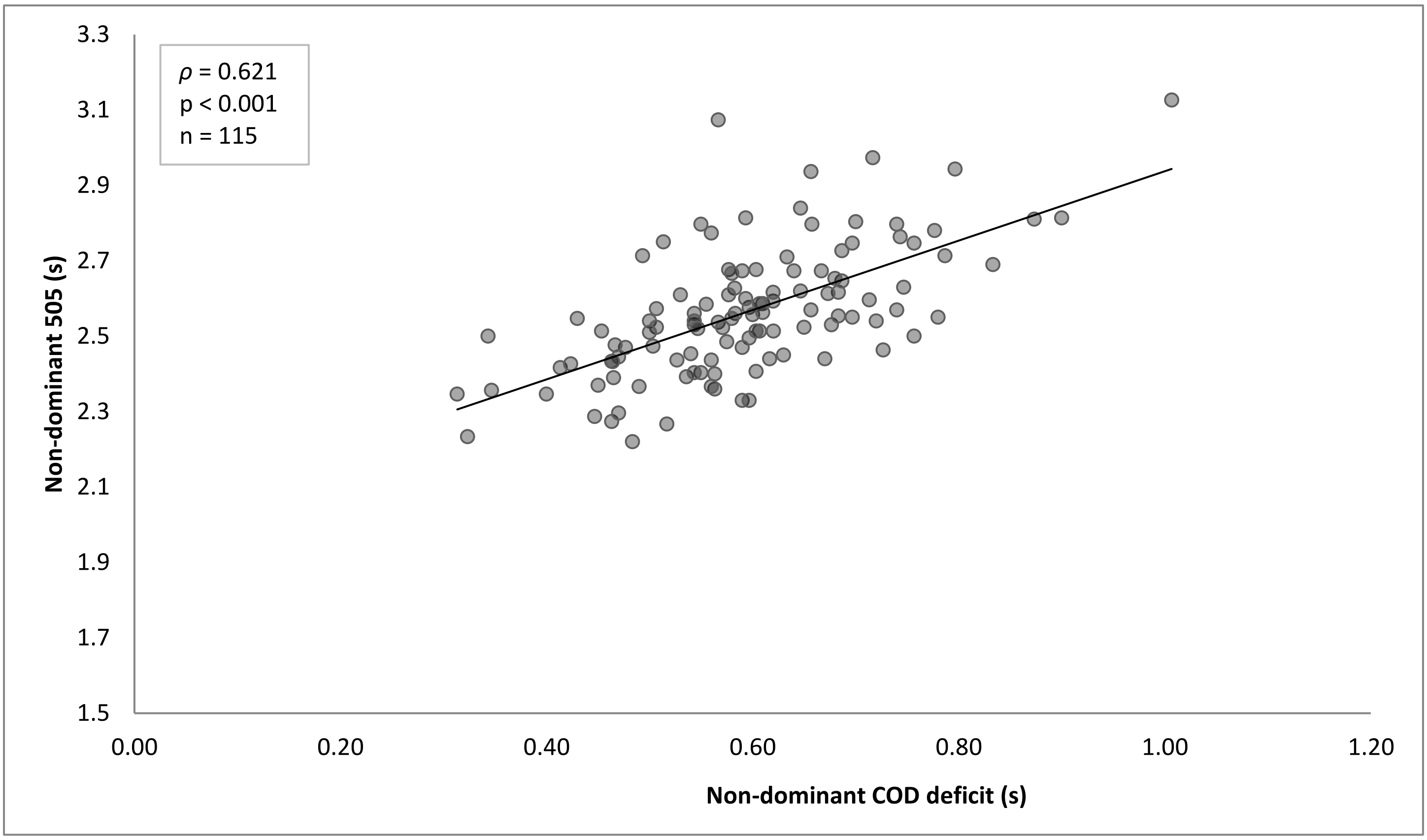

ρ = 0.621
p < 0.001
n = 115
Non-dominant 505 (s)
Non-dominant COD deficit (s)
3.3
3.1
2.9
2.7
2.5
2.3
2.1
1.9
1.7
1.5
0.00
0.20
0.40
0.60
0.80
1.00
1.20

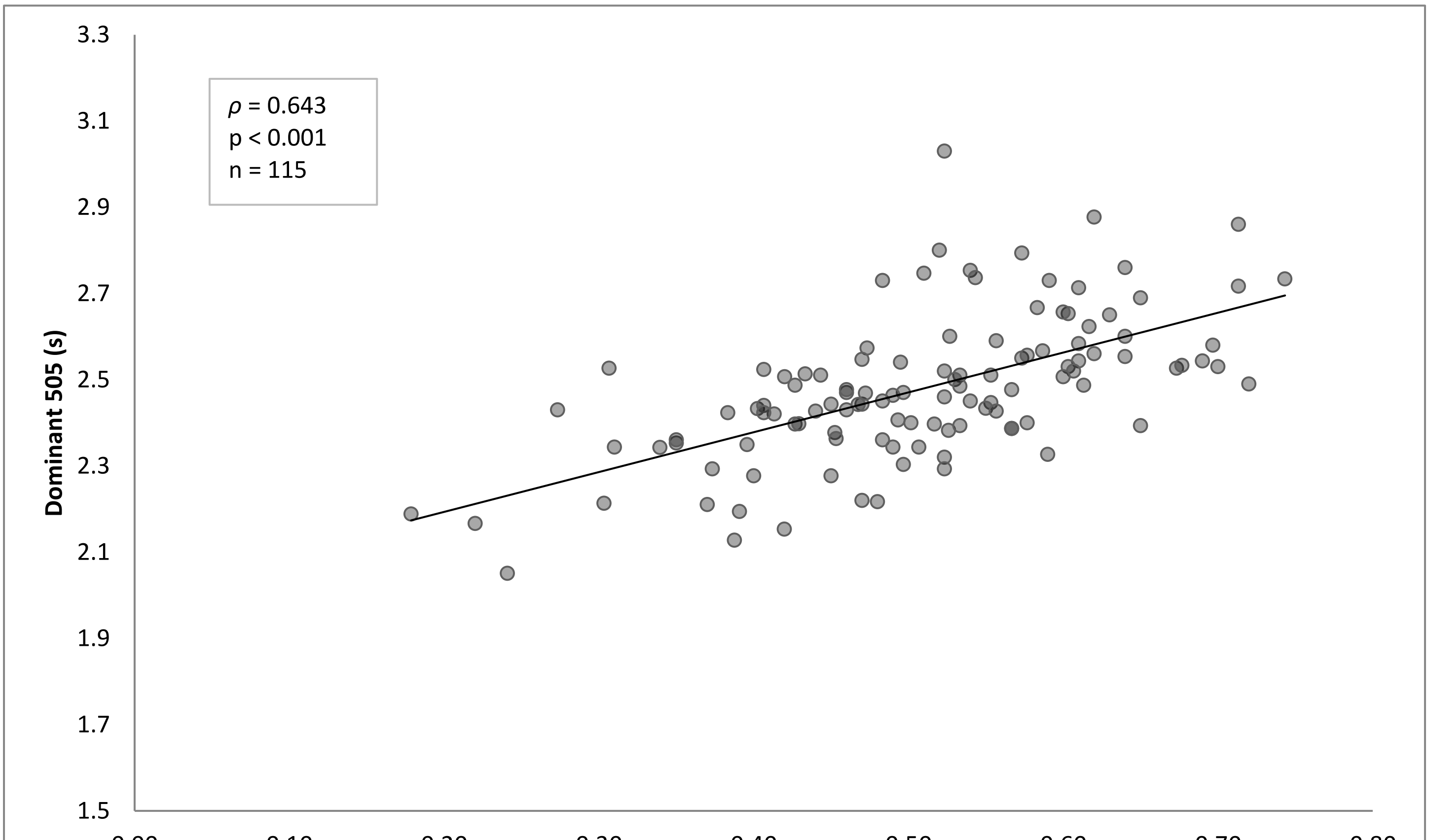

ρ = 0.643
p < 0.001
n = 115
Dominant 505 (s)
Dominant COD deficit (s)
3.3
3.1
2.9
2.7
2.5
2.3
2.1
1.9
1.7
1.5
0.00
0.10
0.20
0.30
0.40
0.50
0.60
0.70
0.80
