# Supplementary material for: Comparison of Change of Direction Speed Performance and Asymmetries between Team-Sport Athletes: Application of Change of Direction Deficit
Source: Sports (Basel). 2018 Dec 12;6(4):174. doi: 10.3390/sports6040174 (PMC6315619; doi:10.3390/sports6040174)
Supplement: Supplementary file 1 [file sports-06-00174-s001.zip › supplementary/Supplementary material 1. updated pairwise .pdf]

Table S1 - Sex and sport descriptive data for 10-m sprint, 505 times, change of direction deficits, and asymmetries between sports

	Pairwise comparisons	10-m sprint (s)		COD deficit D (s)		COD deficit ND (s)		505 D (s)		505 ND (s)		COD deficit imbalance %		505 imbalance %	
		Mean	*SD*	*Mean*	*SD*	*Mean*	*SD*	*Mean*	*SD*	*Mean*	*SD*	*Mean*	*SD*	*Mean*	*SD*
Sex comparison	Male	1.889	0.090	0.497	0.124	0.596	0.126	2.386	0.135	2.484	0.139	-23.2	22.4	-4.2	3.2
	Female	2.047	0.120	0.520	0.096	0.593	0.112	2.567	0.147	2.640	0.165	-14.9	13.3	-2.8	2.4
	Cricket	1.971	0.129	0.524	0.116	0.612	0.098	2.495	0.188	2.584	0.160	-20.4	21.1	-3.7	2.8
Sport comparisons	Court	1.917	0.088	0.533	0.103	0.608	0.125	2.450	0.101	2.525	0.125	-15.3	17.8	-3.0	3.2
	Soccer	2.032	0.157	0.458	0.099	0.552	0.131	2.490	0.198	2.584	0.225	-21.2	15.5	-3.7	2.7

Key: D: Dominant; ND: Non-dominant; COD: Change of direction; F: Female; M: Male

Table S1 - Sex and sport pairwise comparisons in 10-m sprint, 505 times, change of direction deficits, and asymmetries between sports

	Pairwise comparisons	10-m sprint		COD deficit D		COD deficit ND		505 D		505 ND		COD deficit imbalance		505 imbalance	
		p	*g*	*p*	*g*	*p*	*g*	*p*	*g*	*p*	*g*	*p*	*g*	*p*	*g*
Sex comparison	Male vs Female	<0.001	-1.48	0.261	-0.21	0.927	0.02	<0.001	-1.28	<0.001	-1.02	*0.051*	-0.45	0.026	-0.465
	Cricket vs court	0.014	0.48	1.000	-0.09	1.000	0.04	0.325	0.29	0.177	0.41	.287	-0.26	0.325	-0.21
Sport comparisons	Cricket vs soccer	0.012	-0.42	0.027	0.60	0.078	0.52	1.000	0.02	1.000	0.00	1.00	0.04	1.000	0.02
	Court vs soccer	<0.001	-0.89	0.010	0.74	0.099	0.43	0.457	-0.25	0.193	-0.32	.212	0.35	0.457	0.23

Key: D: Dominant; ND: Non-dominant; COD: Change of direction; F: Female; M: Male

Table S1 - Pairwise comparisons between sexes of the same sport for 10-m sprint, 505 times, change of direction deficits, and asymmetries between sports

Pairwise comparisons	10-m sprint		COD deficit D		COD deficit ND		505 D		505 ND		COD deficit imbalance		505 imbalance	
	p	*g*	*p*	*g*	*p*	*g*	*p*	*g*	*p*	*g*	*p*	*g*	*p*	*g*
F cricket vs M cricket	<0.001	1.81	0.524	0.58	1.000	0.22	<0.001	1.64	<0.001	1.53	0.032	0.80	0.030	0.84
F netball vs M basketball	0.005	1.62	1.000	-0.44	0.220	-0.75	1.000	0.67	1.000	0.17	0.234	0.51	0.123	0.60
F soccer vs M soccer	<0.001	1.69	1.000	0.34	1.000	0.49	<0.001	1.48	<0.001	1.45	0.453	-0.35	0.635	-0.31

Key: D: Dominant; ND: Non-dominant; COD: Change of direction; F: Female; M: Male

Table S1 - Pairwise comparisons in 10-m sprint, 505 times, change of direction deficits, and asymmetries between team-sports

Pairwise comparisons	10-m sprint		COD deficit D		COD deficit ND		505 D		505 ND		COD deficit imbalance		505 imbalance	
	p	*g*	*p*	*g*	*p*	*g*	*p*	*g*	*p*	*g*	*p*	*g*	*p*	*g*
F cricket vs F netball	0.029	1.07	1.000	0.50	1.000	0.59	0.019	1.29	0.013	1.30	0.474	-0.14	0.296	-0.21
F cricket vs F soccer	0.177	-0.64	0.338	0.79	1.000	0.29	1.000	0.00	1.000	-0.23	0.041	0.79	0.152	0.59
F cricket vs M basketball	<0.001	2.30	1.000	-0.02	1.000	-0.28	<0.001	1.64	0.005	1.17	0.485	0.45	0.374	0.49
F cricket vs M cricket	<0.001	1.81	0.524	0.58	1.000	0.22	<0.001	1.64	<0.001	1.53	0.032	0.80	0.030	0.84
F cricket vs M soccer	0.001	1.30	0.015	1.23	0.093	0.98	<0.001	2.04	<0.001	1.73	0.116	0.56	0.242	0.39
F netball vs F soccer	<0.001	-1.53	1.000	0.38	1.000	-0.15	0.059	-0.88	0.002	-1.09	0.017	0.86	0.038	0.72
F netball vs M basketball	0.005	1.62	1.000	-0.44	0.220	-0.75	1.000	0.67	1.000	0.17	0.234	0.51	0.123	0.60
F netball vs M cricket	0.056	1.05	1.000	0.20	1.000	-0.37	0.152	0.82	1.000	0.43	0.009	0.85	0.005	0.96
F netball vs M soccer	1.000	0.44	0.655	0.80	1.000	0.45	0.271	1.13	1.000	0.68	0.034	0.65	0.061	0.56
F soccer vs M basketball	<0.001	2.46	0.425	-0.70	1.000	-0.48	0.001	1.21	0.001	1.08	0.265	-0.16	0.533	-0.02
F soccer vs M cricket	<0.001	2.10	1.000	-0.12	1.000	-0.12	<0.001	1.30	<0.001	1.30	0.964	0.19	0.502	0.19
F soccer vs M soccer	<0.001	1.69	1.000	0.34	1.000	0.49	<0.001	1.48	<0.001	1.45	0.453	-0.35	0.635	-0.31
M basketball vs M cricket	1.000	-0.35	0.674	0.53	1.000	0.45	1.000	0.27	1.000	0.19	0.234	0.31	0.331	0.19
M basketball vs M soccer	0.292	-0.91	0.026	1.06	0.011	1.06	1.000	0.35	1.000	0.40	0.471	-0.11	0.829	-0.25
M cricket vs M soccer	1.000	-0.52	1.000	0.43	0.455	0.78	1.000	0.01	1.000	0.24	0.424	-0.47	0.199	-0.55

Key: D: Dominant; ND: Non-dominant; COD: Change of direction; F: Female; M: Male
